# Effect of Lowering Asymmetric Dimethylarginine (ADMA) on Vascular Pathology in Atherosclerotic ApoE-Deficient Mice with Reduced Renal Mass

**DOI:** 10.3390/ijms15045522

**Published:** 2014-03-31

**Authors:** Johannes Jacobi, Renke Maas, Michaela Arend, Nada Cordasic, Karl F. Hilgers

**Affiliations:** 1 Department of Nephrology and Hypertension, Friedrich-AlexanderUniversity Erlangen-Nürnberg (FAU), 91054 Erlangen, Germany; E-Mails: michaela.arend@uk-erlangen.de (M.A.); nada.cordasic@uk-erlangen.de (N.C.); karl.hilgers@uk-erlangen.de (K.F.H.); 2 Institute of Experimental and Clinical Pharmacology and Toxicology, Friedrich-Alexander-University Erlangen-Nürnberg (FAU), 91054 Erlangen, Germany; E-Mail: renke.maas@pharmakologie.med.uni-erlangen.de

**Keywords:** ADMA, subtotal nephrectomy, atherosclerosis

## Abstract

The purpose of the work was to study the impact of the endogenous nitric oxide synthase (NOS) inhibitor asymmetric dimethylarginine (ADMA) and its degrading enzyme, dimethylarginine dimethylaminohydrolase (DDAH1), on atherosclerosis in subtotally nephrectomized (SNX) ApoE-deficient mice. Male DDAH1 transgenic mice (TG, *n* = 39) and C57Bl/6J wild-type littermates (WT, *n* = 27) with or without the deletion of the ApoE gene underwent SNX at the age of eight weeks. Animals were sacrificed at 12 months of age, and blood chemistry, as well as the extent of atherosclerosis within the entire aorta were analyzed. Sham treated (no renal mass reduction) ApoE-competent DDAH1 transgenic and wild-type littermates (*n* = 11) served as a control group. Overexpression of DDAH1 was associated with significantly lower ADMA levels in all treatment groups. Surprisingly, SNX mice did not exhibit higher ADMA levels compared to sham treated control mice. Furthermore, the degree of atherosclerosis in ApoE-deficient mice with SNX was similar in mice with or without overexpression of DDAH1. Overexpression of the ADMA degrading enzyme, DDAH1, did not ameliorate atherosclerosis in ApoE-deficient SNX mice. Furthermore, SNX in mice had no impact on ADMA levels, suggesting a minor role of this molecule in chronic kidney disease (CKD) in this mouse model.

## 1. Introduction

Asymmetrical dimethylarginine (ADMA), an endogenous inhibitor of nitric oxide synthase (NOS), is increasingly recognized as a novel biomarker in cardiovascular disease [1]. The molecule competes with the amino acid, l-arginine, as substrate for all three NOS enzymes and, thereby, limits NO bioavailability. All major known cardiovascular risk factors have been linked to elevated ADMA levels [1]. In addition, various genetic mouse models that modulate ADMA plasma levels emphasize a fundamental role of this NOS inhibitor in vascular pathobiology [2–6].

ADMA does not derive from direct methylation of free l-arginine, but is rather generated from the posttranslational methylation of l-arginine residues within proteins catalyzed by a family of enzymes, called protein arginine methyltransferases (PRMT) [7]. To date, nine PRMT genes have been cloned (PRMT1–9). Based on substrate and product specificity, PRMTs are divided into enzymes with Type I or Type II activity. Both types form monomethyl l-arginine (l-NMMA), but differ in that Type I enzymes produce ADMA, whereas Type II enzymes generate symmetrical dimethylarginine (SDMA) [7]. Upon proteolysis, methylarginines are released into the circulation, where ADMA competes with l-arginine for NOS. ADMA is in part cleared from the circulation via renal excretion and hepatic metabolism; however, the vast majority is metabolized via enzymatic degradation through an enzyme called dimethylarginine dimethylaminohydrolase (DDAH). This enzyme, of which two isoforms with distinct tissue distribution have been identified, catalyzes the hydrolysis of l-NMMA and ADMA into l-citrulline and mono- or di-methylamine [8].

Based on the generation and metabolism of ADMA, elevated levels are the consequence of increased synthesis (enhanced activity or expression of PRMTs), reduced renal clearance or reduced enzymatic degradation (decreased activity or expression of DDAH). The latter two mechanisms have been shown to contribute to elevations of ADMA in renal disease, a condition in which ADMA seems to accumulate [9–12]. In the last few decades, a variety of studies investigated the role of ADMA in patients with different stages of chronic kidney disease (CKD), as well as patients with end-stage renal disease (ESRD). Most studies demonstrate a marked increase of ADMA in patients with renal failure, although the range of reported ADMA levels is wide and overlaps with plasma levels that have been measured in healthy individuals [13].

We have recently generated and characterized transgenic (TG) mice that overexpress the human Isoform 1 of DDAH under control of a β-actin promoter in C57Bl/6J mice [2,5,14]. These animals have lower ADMA levels and an enhanced tissue NOS and DDAH enzyme activity [2]. In a mouse model of atherosclerosis (ApoE-deficient mice), DDAH1 overexpression ameliorated atherosclerotic lesion formation and vascular endothelial function within the aorta [5].

In the present study, we assessed the impact of CKD on the ADMA/DDAH axis in atherosclerosis by studying atherosclerotic lesion formation in subtotally nephrectomized (SNX) ApoE-deficient mice with or without overexpression of DDAH1. We hypothesized that mice with CKD display elevated ADMA levels and that DDAH1 overexpression abrogates atherosclerotic lesion formation in this mouse model.

## 2. Results

### 2.1. Animal Data and Laboratory Chemistry

In all of the three treatment groups, DDAH1 TG mice had a higher body weight at the time point of sacrifice (ANOVA *p* = 0.043, Table 1). In mice with SNX, remnant kidney weight did not differ between WT and TG mice. Blood pressure measured via carotid artery catheters did not differ between treatment groups and genotypes. Creatinine and urea were only mildly, yet significantly, elevated in SNX mice (Table 1). However, histological analysis of remnant kidneys revealed marked glomerulosclerosis as evidenced by markedly elevated Collagen IV deposition (Table 1). In addition, marked glomerular hypertrophy was noted in subtotally nephrectomized mice.

As expected, cholesterol, as well as triglyceride levels were significantly elevated in ApoE-deficient mice of Group 3. Animals in this group had a lower serum albumin compared to mice from other groups (Table 1).

Arterial blood gases obtained at sacrifice were similar between sham treated *versus* SNX mice and across different genotypes, except for lower hemoglobin and hematocrit in SNX mice (Table 2).

### 2.2. Measurement of Methylarginines and l-Arginine

**Figure 1.**
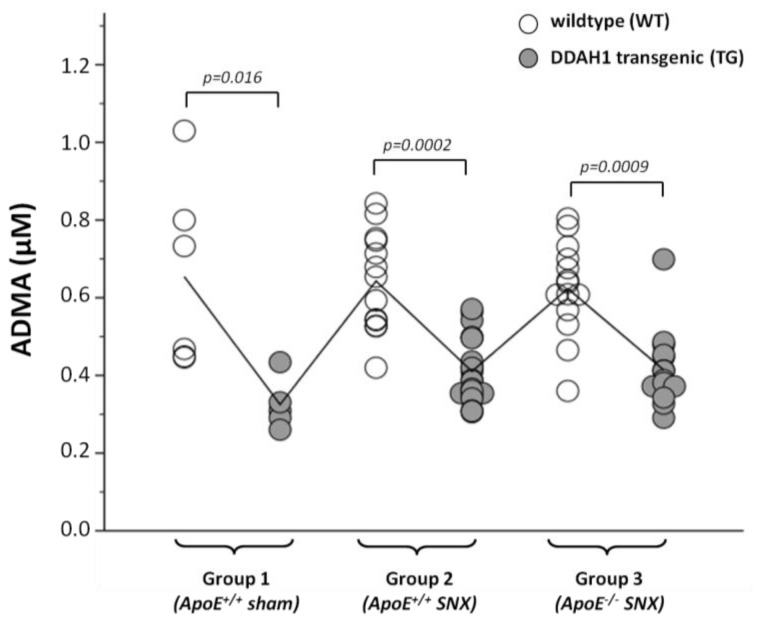
Plasma asymmetric dimethylarginine (ADMA) levels (LC-MS) in mice of different treatment groups. The interpolation line delineates the mean value in each treatment group.

**Figure 2.**
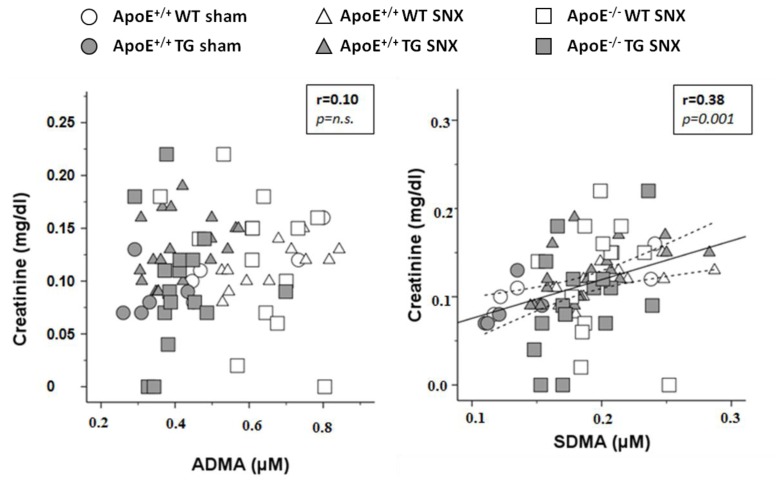
Scatterplots showing the correlation between serum creatinine levels and ADMA (**left panel**) and SDMA (**right panel**).

In all of the three treatment groups, mice overexpressing DDAH1 had markedly lower plasma ADMA levels (35%–50% lower *versus* wild-type (WT) mice; Table 1, Figure 1). Intriguingly, neither SNX nor ApoE-deficiency with SNX was associated with elevated ADMA levels. Thus, within WT and TG mice of different treatment groups, ADMA levels were very similar (Table 1, Figure 1). SDMA levels were only slightly elevated in SNX mice, and the increase was only observed in DDAH1 TG mice that overall had lower SDMA levels than respective WT littermates (Table 1). In WT mice, SDMA levels were similar in sham *versus* SNX treated mice (Table 1). Plasma l-arginine levels did not differ between treatment groups and genotypes. Accordingly, the l-arginine/ADMA ratio was significantly elevated in mice overexpressing DDAH1. Since SDMA is not metabolized by DDAH enzymes, we observed a significant correlation between serum creatinine levels and SDMA, but not ADMA (Figure 2). As previously shown [14], ADMA levels measured by the use of an ELISA assay were slightly higher compared to those obtained with LC-MS (see Table 1). Overall, there was a robust correlation of ADMA levels detected with either method (*r* = 0.82, *p* = 0.0001).

### 2.3. En Face Preparations of the Aorta

**Figure 3.**
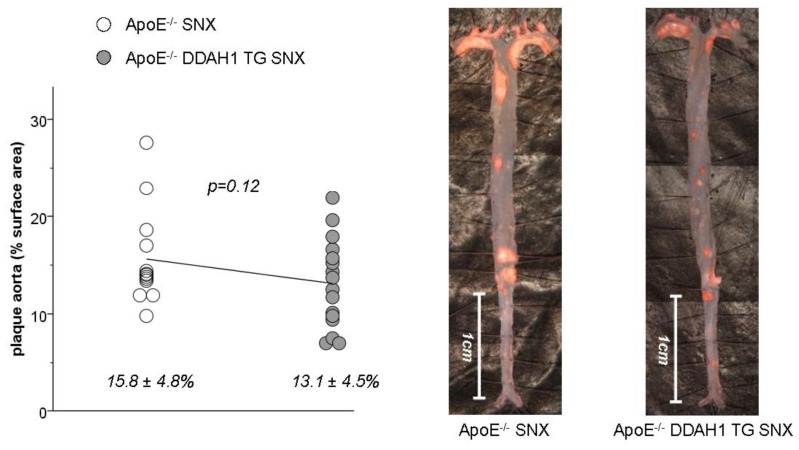
Atherosclerosis within the aorta of ApoE-deficient SNX mice with (grey circles) or without (white circles) overexpression of DDAH1 (**left panel**); Respective Sudan IV stained en face preparations of the aorta (**right panel**).

None of the animals of Group 1 or 2 (ApoE-competent mice with or without SNX) developed atherosclerotic lesions within the aorta (data not shown). In ApoE-deficient SNX mice, the degree of plaque formation within the aorta tended to be somewhat lower in DDAH1 overexpressing mice; however, the difference did not reach statistical significance (Figure 3).

**Figure 4.**
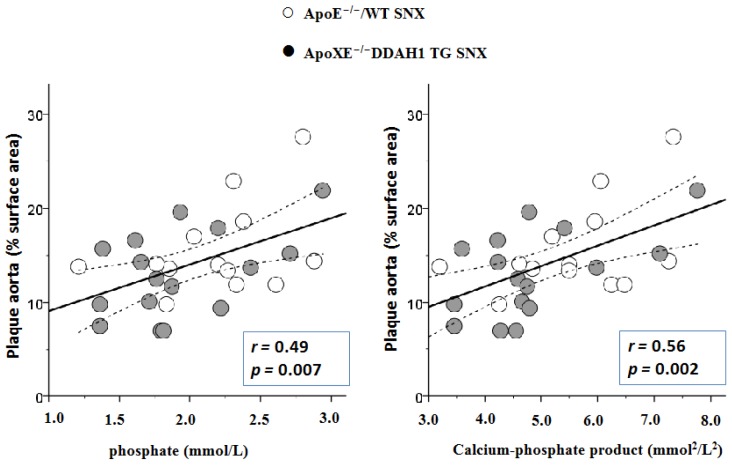
Scatterplots showing the correlation between atherosclerosis and phosphate and the calcium-phosphate product in mice of Group 3.

There was a robust correlation between phosphate levels and calcium-phosphate product and atherosclerosis in ApoE-deficient SNX mice (Figure 4).

### 2.4. Histomorphometry and Immunohistochemistry of the Brachiocephalic Trunk

**Figure 5.**
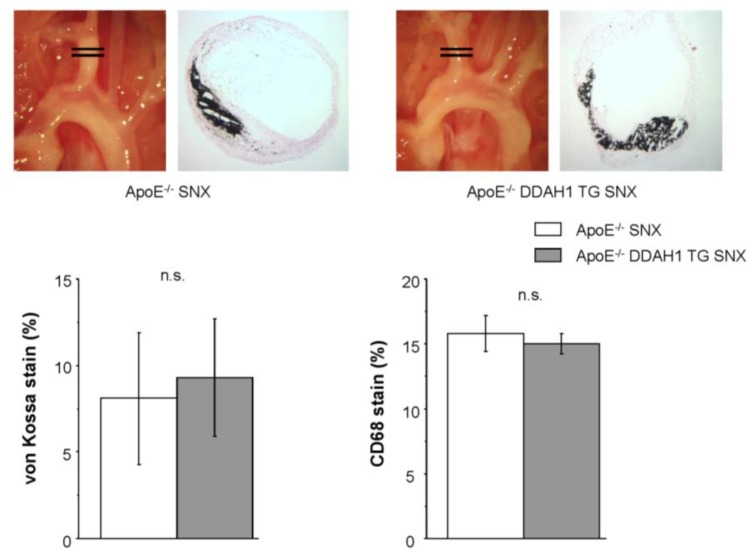
Atherosclerosis and plaque morphology of the brachiocephalic trunk in ApoE-deficient SNX mice. (**Upper panel**) Respective stainings for vascular calcification; (**Lower panel**) quantification of calcified lesions and vascular inflammation.

Cross-sectional analysis of plaques within the brachiocephalic trunk in mice of Group 3 revealed no apparent differences in plaque morphology or plaque composition between WT and TG mice. In particular, luminal narrowing, medial thickening, as well as vascular calcification (von Kossa staining) and inflammation (CD68 immunohistochemistry) were similar between the two genotypes (Figure 5).

### 2.5. Discussion

The salient findings of the present study are as follows: (1) overexpression of the ADMA degrading enzyme, DDAH1, does not reduce plaque formation in SNX treated ApoE-deficient mice; and (2) CKD in mice induced by SNX does not affect plasma levels of ADMA.

The former observation stands in contrast to results obtained in age- and sex-matched ApoE-deficient mice without CKD that we have previously published [5]. In these studies, the overexpression of DDAH1 reduced atherosclerotic lesion formation and improved vascular endothelial function. In the present study, DDAH1 overexpression only affords a minor, yet not significant, benefit with regard to plaque formation in the aorta, indicating a less important role of this enzyme in this mouse model under uremic conditions. Notably, plasma levels of both ADMA and SDMA in the present experiments were very similar to those observed in mice without renal ablation [5].

The latter finding, namely the lack of an effect of SNX or CKD on plasma levels of ADMA, is quite unexpected given previous findings on ADMA and SDMA in CKD. In contrast to SDMA, renal excretion plays only a minor, yet not precisely known, role in ADMA clearance. The major metabolism occurs via degradation through DDAH enzymes, of which two isoforms have been identified [8,15]. Recent evidence suggests that DDAH1 is the critical enzyme for ADMA degradation [4]. In addition, recent studies have identified the enzyme, alanine-glyoxylate aminotransferase-2 (AGXT2), as a major source of degradation of methylarginines [16]. In contrast to DDAH, AGXT2 also metabolizes SDMA. The somewhat lower SDMA levels in DDAH1 TG mice observed in the present study may result from greater metabolism through AGXT2 in the presence of low ADMA levels. This hypothesis is currently tested in ongoing experiments.

Given the clear-cut effect of DDAH1 overexpression on plasma ADMA levels in all three treatment groups, we feel very confident that the lack of an effect of SNX on the plasma levels of ADMA does not reflect an analytical error, but is indeed a true and valid finding. However, we cannot entirely rule out that renal mass reduction was insufficient to produce advanced CKD. However, our long lasting experience with this experimental animal model shows marked perioperative mortality after more radical renal mass reduction. Of note, we deliberately wanted to study the long-term effects of CKD on vascular pathobiology and, thereby, refrained from using an atherosclerotic diet or an earlier time point of sacrifice. Thus, although SNX mice were not hypertensive and had a normal acid base homeostasis, they had significantly elevated creatinine and urea levels and were anemic.

The lack of an effect of SNX on plasma ADMA levels stands in contrast with recent reports by others in which SNX in mice were associated with a ~20% increase of plasma ADMA levels [17,18]. In both studies, mean plasma ADMA levels increased from approximately 1.0 to 1.2 μM. Of note, plasma ADMA levels were significantly lower in our studies, the highest plasma ADMA level was 1.03 μM and was measured in an ApoE-competent WT animal without SNX. Using the same DDAH1 TG mouse model, Kajimoto *et al.* observed a ~20% increase of ADMA levels in SNX treated WT mice, whereas in TG mice, no significant change of ADMA was seen [18]. The effect of the transgene on ADMA levels was comparable to the one observed in the present study. Unfortunately, both studies lack information on plasma SDMA levels [17,18]. While we cannot fully explain these discrepant findings, we speculate that the different timeframe of the experiments may serve as an explanation. Notably, we studied the long-term effects in a very chronic model or reduced kidney function nine months after 5/6 nephrectomy, whereas others investigated mice only one month after nephrectomy [17,18]. During our longer observation period, molecular mechanisms responsible for either the formation of ADMA (e.g., PRMT enzymes) or its metabolism (e.g., DDAH) may have undergone adaptive changes to counteract an increase of ADMA levels. Further research will be necessary to explore these possibilities. Nevertheless, the lack of increased ADMA levels in nephrectomized mice in our experiments limits the conclusions of our study.

Although calcium and phosphate levels were not significantly elevated in ApoE-deficient SNX mice, we observed a striking correlation between phosphate and the calcium phosphate product and atherosclerosis. These observations are compatible with the known role of mineral bone disease on atherosclerosis in CKD [19].

The effect of calcium-phosphate on atherosclerosis and vascular calcification seems to be driven by phosphate levels, as calcium levels alone did not correlate with atherosclerosis in our study. This may help to explain why calcium carbonate supplementation that is used as a phosphate binder in patients with CKD does not promote vascular calcification in uremic ApoE-deficient mice [20]. In contrast, the same authors could demonstrate that the calcimimetic R-568 lowers both calcium and phosphate in this animal model, and this effect is accompanied by less atherosclerosis and vascular calcification [21]. In the present study, vascular calcification within atherosclerotic lesions of the brachiocephalic trunk was only slightly more pronounced compared to previously published studies in ApoE-deficient mice without renal ablation [5].

Another important observation of this study is the fact that the degree of atherosclerosis in SNX treated ApoE-deficient mice of Group 3 is only marginally higher compared to our previously published data in age- and sex-matched ApoE-deficient mice without renal ablation [5]. Thus, in the present study, the average plaque content in the entire aorta of *n* = 13 SNX ApoE-deficient mice (see Figure 2) is 15.8% compared to 12.4% in our previous study in *n* = 15 ApoE-deficient mice without CKD [5]. This observation stands in sharp contrast to previous reports by others that describe the acceleration of atherosclerosis in uremia using ApoE-deficient mice [22–24].

However, there are several aspects that may explain the observed discrepancies. First of all, differences may be related to the genetic background of ApoE-deficient mice or differences in rodent diet. Accordingly, our mice were purchased from Jackson Laboratories, whereas the other investigators used different vendors. Furthermore, the study by Buzello *et al.* [23] used a cholesterol diet that may accelerate lesion formation under uremic conditions. More importantly, as mentioned above, the duration of CKD was shorter in all of the three studies and varied between six and 22 weeks after the induction of SNX. In addition, in the studies of Massy *et al.*, electrocautery was used to achieve renal mass reduction [24]. Finally, we meticulously analyzed atherosclerosis by en face preparations spanning the entire aorta, whereas previous studies focused on cross-sectional analysis of lesion formation in the ascending aorta. As can be seen from the images of Figure 4, the degree of atherosclerosis within this specific anatomical region can vary considerably between animals, while the overall plaque content within the entire aorta is rather similar. This observation prompted us to study cross-sectional lesions within the distal brachiocephalic trunk, where we noted far less variation in plaque deposition.

We are on the way to further study the role of methylarginines in CKD in various genetic mouse models that will hopefully help to address many of the unmet issues and questions raised by the current experiments.

## 3. Experimental Section

### Animals

Human DDAH1 TG mice (hDDAH1^+/−^) were generated, maintained and genotyped as previously described in detail [5]. ApoE-deficient mice (ApoE^−/−^) were purchased from Jackson Laboratories (Apoe^tm1Unc^, stock Number: 002052) and were backcrossed into C57Bl/6J background for ten generations.

Genotyping was performed according to the PCR protocol provided by the vendor.

ApoE^−/−^/hDDAH1^+/−^ mice were generated as previously described [5].

All animals were housed in a temperature-controlled animal facility with a 12-h light/dark cycle and free access to tap water and standard rodent chow. Mice were sacrificed in the morning hours during 08:00–12:00 am.

The study protocol was approved by the animal research ethics committee of the local government (Bezirksregierung Mittelfranken, AZ 54-2531.31-1/06).

## 4. Experimental Groups

### 4.1. Three Groups of Mice Were Studied (Total n = 77)

Group 1 (ApoE^+/+^ control group without SNX, *n* = 11) was ApoE^+/+^ male mice with (TG, *n* = 5) or without (WT, *n* = 6) overexpression of hDDAH1.

Group 2 (ApoE^+/+^ mice with SNX, *n* = 36) was ApoE^+/+^ SNX male mice with (TG, *n* = 23) or without (WT, *n* = 13) overexpression of hDDAH1.

Group 3 (ApoE^−/−^ mice with SNX, *n* = 30) was ApoE^−/−^ SNX male mice with (TG, *n* = 16) or without (WT, *n* = 14) overexpression of hDDAH1.

All mice were sacrificed at the age of 12 months.

### 4.2. Subtotal Nephrectomy (SNX)

Mice of experimental Groups 2 and 3 underwent SNX. Briefly, at 8 weeks of age, a right-sided nephrectomy or sham surgery (Group 1) was performed via a flank incision. Two weeks later, the upper and lower thirds of the left kidney were resected and weighed to achieve SNX, or a sham procedure (Group 1) was performed.

### 4.3. Blood Pressure Measurements

Before sacrifice, blood pressure was measured via carotid artery catheters in conscious mice. Briefly, the right carotid artery was exposed and cannulated with a polyethylene tubing catheter under inhalation anesthesia (isoflurane 2%). The catheter was secured with sutures and subcutaneously tunneled to exit at the back of the neck. The end of the catheter was connected to a Statham pressure transducer and a Gould polygraph. After recovery from the surgical procedure, blood pressure was measured in conscious, restrained animals over 20 min.

### 4.4. Laboratory Tests

After blood pressure recordings, animals were exsanguinated under inhalation anesthesia (2% isoflurane) via the carotid artery catheters. The following parameters were measured: arterial blood gases, creatinine, urea, total protein, albumin, phosphate, cholesterol, triglycerides, ADMA, SDMA and l-arginine.

### 4.5. Measurement of Methylarginines and l-Arginine

Plasma levels of l-arginine, ADMA, as well as symmetric dimethylarginine (SDMA) were measured by use of liquid chromatographic-tandem mass spectrometry, as previously described [14]. In addition, ADMA was also measured by use of a commercially available ELISA assay, as previously described [14]. Samples were measured in duplicate, and mean values were computed; the intra-assay coefficient of variation was <6%.

### 4.6. En Face Preparations of the Aorta

Atherosclerosis within the aorta was analyzed in mice of Group 3 (*n* = 30) by performing Sudan IV stained en face preparations spanning the entire aorta from the aortic root to the iliac bifurcation, as recently described in detail [5].

Native, as well as Sudan IV stained preparations were captured with a digital camera attached to the microscope. Atherosclerotic lesions were quantified by using image analysis software (MetaVue version 4.6r9; Molecular Devices, Sunnyvale, CA, USA), and the lesion area was expressed as a percentage of total aortic surface area.

### 4.7. Histomorphometry and Immunohistochemistry of the Brachiocephalic Trunk and Remnant Kidneys

Cross-sectional lesion formation was analyzed within the distal portion of the brachiocephalic trunk. Cryostat cross-sections (8 μm) were stained with H&E or PAS, von Kossa (calcification), as well as CD68 (macrophage infiltration). Controls included omission of primary antibodies.

In order to confirm chronic kidney disease in mice with renal ablation, glomerular diameters and Collagen IV deposition were measured in kidney cross-sections from mice of all treatment groups. Briefly, glomerular diameter was measured in 100 glomeruli using image analysis software (MetaVue version 4.6r9; Molecular Devices, Sunnyvale, CA, USA). Glomerular Collagen IV staining (1:500; Southern Biotechnology, Birmingham, AL, USA) was measured in 30 randomly chosen glomeruli per cross-section. The stained area was expressed relative to the total area of the glomerular tuft.

### 4.8. Statistical Analysis

Statistical analysis was performed by using the SPSS software package (version 16.0; SPSS Inc., Chicago, IL, USA). Differences between WT and TG mice in each subgroup were first analyzed by using the unpaired *t*-test. Differences between the three study groups were further analyzed by using one-way analysis of variance with post hoc Bonferroni correction. In addition, a univariate linear model was used to analyze the effects of SNX, as well as DDAH1 or ApoE genotype throughout the three treatment groups. Pearson correlation coefficients were calculated when indicated. All data are given as the mean ± SD. Statistical significance was accepted at a value of *p* < 0.05 (two-sided).

## 5. Conclusions

In conclusion, plasma levels of ADMA are unaltered in our SNX model of CKD in mice. In addition, overexpression of the ADMA degrading enzyme, DDAH1, in SNX treated ApoE-deficient mice does not ameliorate atherosclerosis. Our data clearly do not support the notion that ADMA plays a particularly important role for atherosclerosis in CKD.

## Figures and Tables

**Table 1.** Characteristics and laboratory chemistry of mice. SNX, subtotally nephrectomized; WT, wild-type; TG, transgenic.

	ApoE^+/+^ WT sham (*n* = 6)	ApoE^+/+^ DDAH1 TG sham (*n* = 5)	ApoE^+/+^ WT SNX (*n* = 13)	ApoE^+/+^ DDAH1 TG SNX (*n* = 23)	ApoE^−/−^ WT SNX (*n* = 14)	ApoE^−/−^ DDAH1 TG SNX (*n* = 16)	ANOVA *p*-value	DDAH1 effect *p*-value	SNX effect *p*-value	ApoE effect *p*-value
weight (g)	31.6 ± 1.2	32.3 ± 1.0	30.2 ± 1.9	31.3 ± 1.9	30.5 ± 2.6	31.8 ± 2.4	n.s.	0.043	n.s.	n.s.
kidney weight (mg/g BW)	16.9 ± 1.5	16.8 ± 1.9	7.5 ± 1.1	7.4 ± 1.9	9.0 ± 1.6	8.6 ± 1.0	0.0002	n.s.	0.0001	n.s.
glomerular diameter (μm)	169 ± 15	170 ± 10	195 ± 14	187 ± 17	234 ± 36	224 ± 30	0.0002	n.s.	0.001	0.0007
glomerular collagen 4 (%)	2.24 ± 1.70	3.55 ± 4.31	5.85 ± 3.81	4.78 ± 4.09	26.23 ± 7.68	21.26 ± 5.59	0.0001	n.s.	0.006	0.0005
mean arterial BP (mmHg)*	107 ± 7	111 ± 5	109 ± 5	109 ± 7	102 ± 12	108 ± 12	n.s.	n.s.	n.s.	n.s.
heart rate (beats/min)*	610 ± 35	596 ± 38	594 ± 54	630 ± 36	574 ± 53	610 ± 44	n.s.	n.s.	n.s.	n.s.
creatinine (mg/dL)	0.11 ± 0.03	0.09 ± 0.03	0.12 ± 0.02	0.13 ± 0.03	0.12 ± 0.06	0.10 ± 0.06	n.s.	n.s.	0.010	n.s.
urea (mg/dL)	73.8 ± 11.6	68.1 ± 18.4	87.3 ± 18.0	92.1 ± 15.3	104.7 ± 33.5	102.9 ± 17.7	0.002	n.s.	0.0004	0.001
cholesterol (mg/dL)	78 ± 7	76 ± 15	76 ± 13	80 ± 7	480 ± 142	447 ± 166	0.0002	n.s.	0.011	0.0001
triglycerides (mg/dL)	108 ± 21	81 ± 28	74 ± 42	90 ± 39	138 ± 84	136 ± 72	0.013	n.s.	n.s.	0.0003
total protein (g/L)	46.7 ± 7.4	43.5 ± 7.0	49.0 ± 19.9	48.4 ± 3.3	40.0 ± 14.2	45.0 ± 6.2	n.s.	n.s.	n.s.	n.s.
albumin (g/L)	30.7 ± 3.9	29.5 ± 3.9	32.3 ± 9.4	32.3 ± 2.3	23.6 ± 8.0	27.5 ± 3.7	0.004	n.s.	n.s.	0.0002
phosphate (mmol/L)	1.92 ± 0.28	2.27 ± 0.22	2.02 ± 0.54	1.88 ± 0.27	2.11 ± 0.53	1.92 ± 0.47	n.s.	n.s.	n.s.	n.s.
ADMA ELISA (μmol/L)	0.68 ± 0.33	0.27 ± 0.15	0.66 ± 0.08	0.48 ± 0.15	0.75 ± 0.20	0.59 ± 0.12	0.0008	0.0002	n.s.	n.s.
ADMA (μmol/L)	0.65 ± 0.24	0.32 ± 0.07	0.64 ± 0.13	0.41 ± 0.08	0.62 ± 0.12	0.41 ± 0.09	0.0003	0.0006	n.s.	n.s.
SDMA (μmol/L)	0.20 ± 0.08	0.13 ± 0.02	0.20 ± 0.04	0.19 ± 0.03	0.20 ± 0.02	0.18 ± 0.03	0.007	0.049	0.026	n.s.
l-arginine (μmol/L)	87.9 ± 19.4	92.0 ± 30.2	70.7 ± 35.9	75.4 ± 28.9	68.5 ± 30.4	83.0 ± 24.4	n.s.	n.s.	n.s.	n.s.
l-arginine/ADMA ratio	141 ± 28	278 ± 44	107 ± 42	181 ± 50	111 ± 45	202 ± 48	0.0005	0.0003	0.027	n.s.

BW, body weight; BP, blood pressure; ADMA, asymmetric dimethylarginine; SDMA, symmetric dimethylarginine;

* carotid artery catheter;

n.s. = not significant.

**Table 2.** Arterial blood gas analysis at the time of sacrifice (12 months).

	ApoE^+/+^ WT sham (*n* = 6)	ApoE^+/+^ DDAH1 TG sham (*n* = 5)	ApoE^+/+^ WT SNX (*n* = 13)	ApoE^+/+^ DDAH1 TG SNX (*n* = 23)	ApoE^−/−^ WT SNX (*n* = 14)	ApoE^−/−^ DDAH1 TG SNX (*n* = 16)	ANOVA *p*-value	DDAH effect *p*-value	SNX effect *p*-value	ApoE effect *p*-value
pH	7.37 ± 0.06	7.29 ± 0.02	7.33 ± 0.04	7.37 ± 0.06	7.31 ± 0.07	7.35 ± 0.04	0.011	n.s.	n.s.	n.s.
pO_2_ (mmHg)	121 ± 3	126 ± 11	136 ± 17	130 ± 24	131 ± 17	113 ± 25	n.s.	n.s.	n.s.	n.s.
pCO_2_ (mmHg)	37 ± 4	41 ± 4	37 ± 4	38 ± 8	41 ± 6	38 ± 11	n.s.	n.s.	n.s.	n.s.
BE (mmol/L)	−3.7 ± 1.6	−6.5 ± 2.4	−6.0 ± 3.0	−4.3 ± 4.3	−5.0 ± 4.7	−5.8 ± 3.7	n.s.	n.s.	n.s.	n.s.
bicarbonate (mmol/L)	20.9 ± 1.2	19.1 ± 2.0	19.1 ± 2.7	20.2 ± 4.4	20.0 ± 4.2	19.3 ± 3.2	n.s.	n.s.	n.s.	n.s.
hemoglobin (g/dL)	14.1 ± 0.5	13.8 ± 1.3	13.1 ± 1.2	12.7 ± 1.1	12.1 ± 1.6	12.8 ± 1.3	n.s.	n.s.	0.017	n.s.
hematocrit (%)	43.1 ± 1.5	42.3 ± 3.8	40.7 ± 3.4	39.2 ± 3.3	35.4 ± 7.7	39.5 ± 4.1	0.049	n.s.	0.038	0.030
sodium (mmol/L)	145.2 ± 0.8	146.9 ± 1.6	147.2 ± 2.5	143.3 ± 7.4	145.4 ± 4.1	144.1 ± 5.2	n.s.	n.s.	n.s.	n.s.
potassium (mmol/L)	3.5 ± 0.3	3.6 ± 0.2	3.5 ± 0.5	3.9 ± 0.3	3.9 ± 0.5	3.7 ± 0.4	n.s.	n.s.	n.s.	n.s.
chloride (mmol/L)	115.7 ± 1.2	116.2 ± 0.8	118.9 ± 5.0	116.4 ± 4.5	117.0 ± 2.2	118.6 ± 3.2	n.s.	n.s.	n.s.	n.s.
ion calcium (mmol/L)	1.29 ± 0.02	1.29 ± 0.04	1.31 ± 0.07	1.26 ± 0.05	1.27 ± 0.05	1.26 ± 0.06	n.s.	n.s.	n.s.	n.s.
